# Biosynthesis of Silver Nanoparticles from Hybrid Polymer: Characterization, Approach from XRD and Investigation of Antimicrobial Activity

**DOI:** 10.1021/acsomega.5c12247

**Published:** 2026-04-11

**Authors:** Expedito Lopes Fernandes Júnior, Izabel Maria de Melo Amaral, George Torres de Lima, Raí Emanuel da Silva, Alyne Rodrigues de Araújo Nobre, Rafael Alexandre Raimundo, Luise Lopes Chaves, Antônia Carla de Jesus Oliveira, Teresinha Gonçalves Silva, Mônica Felts de La Roca Soares, José Lamartine Soares Sobrinho, Amanda Damasceno Leão

**Affiliations:** † Quality Control Core of Medicines and Correlates—NCQMC, Department of Sciences, 28116 Federal University of Pernambuco—UFPE, Recife, State of Pernambuco CEP: 50670-901, Brazil; ‡ Laboratory of Pharmatoxicological Prospecting of Biological Products, Department of Antibiotics, Federal University of Pernambuco—UFPE, Recife, State of Pernambuco CEP: 50670-901, Brazil; § Biodiversity and Biotechnology Research Center -BIOTEC, Campus de Parnaíba, Parnaíba Delta Federal University- UFDPar, Parnaíba, State of Piauí CEP: 64202-020, Brazil; ∥ Department of Materials Science and Engineering, 28123 Federal University of Rio Grande do Norte—UFRN, Natal, State of Rio Grande do Norte CEP: 59078-970, Brazil; ⊥ TEMA - Centre for Mechanical Technology and Automation, Department of Mechanical Engineering, University of Aveiro, Aveiro CEP: 3810-193, Portugal

## Abstract

Silver nanoparticles (AgNPs) have recognized antimicrobial activity, but they are potentially harmful when obtained by traditional synthesis. In this context, biosynthesis offers a viable alternative using biological reducing agents. This work reports the biosynthesis of AgNPs using two materials: the biopolymer *cashew gum* (CG) and CG polymerized in situ with poly­(methyl methacrylate) (PMMA). Fourier transform infrared (FTIR) spectroscopy confirmed the polymerization of PMMA/CG by the absence of C=C unsaturation. For both AgNPs, UV–vis absorption spectra confirmed their formation, as evidenced by the appearance of the surface plasmon resonance band. FTIR analysis of the AgNPs indicated that O–H and C=O groups participated in the silver reduction. The more significant reduction of these bands in AgNP-PMMA/CG suggests that the copolymer is an effective reducing agent for AgNP formation. These findings were corroborated by zeta potential measurements, which demonstrated greater colloidal stability for AgNP-PMMA/CG. XRD (X-ray diffractometry) peaks for both AgNPs were in agreement with the Miller indices (*hkl*) and indexed values (Ag—ICSD 22434), confirming a typical face-centered cubic structure. Through Rietveld refinement, a greater microstrain was observed for AgNP-CG (0.015) than for AgNP-PMMA/CG (0.01), despite a slight loss in the precision of the structural fit. AFM analysis of AgNP-PMMA/CG showed a more spherical shape and defined edges. In addition, the size distribution indicated a smaller size and less heterogeneity compared to AgNP-CG. Regarding antimicrobial activity, AgNP-PMMA/CG demonstrated bacteriostatic activity against all tested bacteria, *Pseudomonas aeruginosa*, *Escherichia coli*, and *Staphylococcus aureus*, while AgNP-CG showed activity only against Gram-negative strains. It also showed adequate cell viability in murine macrophages at all concentrations tested. In conclusion, the copolymer obtained was the most effective for the biosynthesis of AgNP-PMMA/CG. This material is considered promising due to its optimal structural organization, antimicrobial activity, biocompatibility, and more sustainable synthesis method.

## Graphical abstract

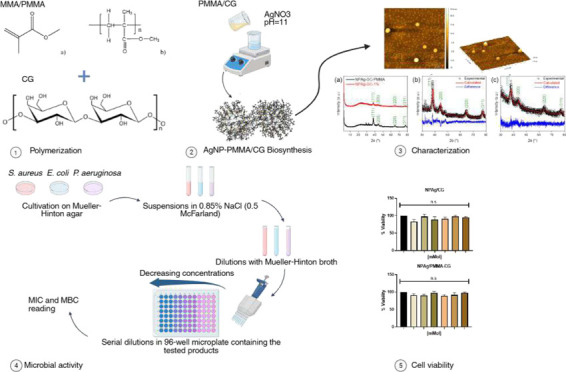

## 1 Introduction

Silver nanoparticles (AgNPs) are nanometric particles formed by nucleation through the reduction of Ag^+^ cations, resulting in colloidal particles stabilized by electrostatic repulsion.^1^ Their antimicrobial potential is particularly valuable in the current context, where self-medication and unregulated prescriptions contribute to the rise of bacterial resistance at a faster rate than new antimicrobials are developed. Meanwhile, infections caused by multidrug-resistant microorganisms continue to increase and result in deaths worldwide.^2^

Furthermore, AgNPs exhibit broad-spectrum antibacterial activity, customizable size and shape, and the ability to penetrate bacterial cell membranes due to their high surface/volume ratio. The mechanism of action of AgNPs reduces the possibility of known resistance mechanisms, as Ag^+^ ions are responsible for their natural antimicrobial activity, presenting themselves as a potential clinical alternative.^3^

However, the traditional chemical synthesis of AgNPs has disadvantages, such as high energy consumption, expensive equipment, and the use of compounds that are toxic to humans and the environment. In this sense, green synthesis, or AgNP biosynthesis, is an ecologically sustainable alternative to traditional techniques. In biosynthesis, microorganisms or substances of biological origin can be used to reduce Ag^+^ cations, which then undergo nucleation. This is an important strategy for enabling the application of AgNPs through more sustainable and biocompatible methods and formulations.^4^

*Cashew gum* (CG) is a vegetable gum and a heteropolysaccharide produced by the plant in response to physical and chemical stimuli. Obtained from the resin extracted from the trunk exudate of *Anacardium occidentale* L., CG has been documented in the literature for its use in the synthesis and optimization of nanometric formulations, such as AgNPs. Furthermore, its chemical modification may allow for the optimization of its functional and structural properties.^5–7^

The structural modification of CG with PMMA poly­(methyl methacrylate) can alter its physicochemical properties, resulting in an improved material. Literature data indicate that the modification of CG with acrylic polymers, such as PMMA, represents one of the most promising possibilities. For example, poly­(acrylamide), poly­(isopropylacrylamide), and poly­(glycidyl methacrylate) have been used in association with CG for applications ranging from the formulation of gels, nanoparticles (NPs), and drug delivery matrices. This approach offers advantages such as higher viscosity, biocompatibility, greater colloidal stability, and increased thermal resistance.^8–11^

PMMA is an acrylic polymer that can be associated with CG due to its superior mechanical properties, such as hardness, rigidity, low toxicity, and long-term mechanical stability after implantation. It is also explored as a suitable base material for biomedical formulations. Its applications include use as a strengthening agent in bone cement, drug delivery systems, and as a raw material for nanofiber formation. In all these applications, PMMA is almost always copolymerized or used in conjunction with other polymers to form polymer blends, which helps to complement the properties of the materials selected.^12–15^

Thus, the PMMA/CG modification combines a biological reducing agent with a synthetic polymer that has been little explored in AgNP synthesis. Both of these components have reducing groups that can contribute to the formation and stabilization of AgNPs. As a result, biosynthesized AgNPs may have applications in the biomedical field, either in liquid form, or dried for incorporation into a polymeric film for local action. This suggests their application as a promising antimicrobial agent due to its suitable structural organization, biocompatibility, and more sustainable synthesis method.

## 2 Materials and Methods

CG was isolated from the tree species *A. occidentale* (*M*_w_ = 1.8 × 10^5^ g mol^–1^) using an adapted method previously described by de Paula et al. (1998),^16^ was purified at the Center for Quality Control of Medicines and Correlates (NCQMC) and provided by GumLife. Commercial MMA was obtained from Sigma-Aldrich, PBZ was obtained from Dinâmica and the other reagents were of analytical grade. Purified water was obtained by reverse osmosis with a Milli-Q system. The glassware and utensils used in the preparation of AgNPs were covered with aluminum foil.

### 2.1 GC/PMMA in Situ Polymerization

MMA was previously partitioned to remove polymerization inhibitors^17^ PMMA/CG were prepared in proportions of 1:1, 1:4, 1:6, and 1:8 (wt wt^–1^). Calculations were made for the volume of MMA and the initiator benzoyl peroxide (PBZ) 1% MMA (wt wt^–1^). Initially, MMA and CG were kept under stirring at room temperature for better dispersion. Then, PBZ was added and the mixture was heated to 80 °C to initiate radical polymerization. The systems were left to rest for 24 h to ensure the reaction’s complete conversion^17^ (Representative diagram in the Supporting Information).

The CG/PMMA systems were solubilized with methyl ethyl ketone (MEK) for 24 h. Then, 4 mL aliquots were transferred to Petri dishes and dried in an oven (40 °C for 4 h) until the complete evaporation of the MEK (casting drying). The resulting film was carefully removed from the plates and crushed in the presence of liquid nitrogen.^17^ The resulting powder was then stored in a desiccator.

### 2.2 Obtaining Silver Nanoparticles by Biosynthesis

Initially, a 0.1% dispersion of PMMA/CG was prepared in ultrapure water. The system was placed under magnetic stirring at 400 rpm and room temperature for 24 h to ensure proper polymer dispersion. Subsequently, 50 mL of a 1 mM silver nitrate (AgNO_3_) solution was prepared, and the pH was adjusted to 11 with a NaOH solution (5 M).^6^

To obtain the NP, 50 mL of the 0.1% copolymer PMMA/CG dispersion was poured into a 1 mmol L^–1^ AgNO_3_ solution under magnetic stirring (400 rpm) and heated (70 °C) for 1 h to reduce the Ag^+^ cations. After centrifugation (15 min/3500 rpm), the NPs were frozen in a freezer (−15 °C) and subsequently lyophilized (−45 °C, 500 mmHg, 98 h).^6^

### 2.3 UV–vis and FTIR Spectroscopies

Ultraviolet–visible (UV–vis) spectroscopy was performed using a UV-1900I instrument, using the range between 300 and 600 nm. Readings were taken at 0.5 h intervals during the biosynthesis of AgNPs. For each reading, a 1 mL aliquot of the reaction suspension was taken and diluted 1:10 (v/v) with water.^6^

Fourier transform infrared spectroscopy (FTIR) was obtained using a PerkinElmer (Spectrum 400) equipment with an attenuated total reflectance (ATR) device with a zinc selenide crystal, obtained from 650 cm^–1^ to 4000 cm^–1^ at a resolution of 4 cm^–1^.^6^

### 2.4 X-ray Diffractometry (XRD)

The analyses were performed at room temperature, in an XRD-6100 diffractometer using Cu radiation, voltage of 40 kV and current of 30 mA. Data acquisition was performed in continuous mode, with simultaneous movement of the detector and the sample holder (step size 0.0120 deg, scanspeed 2.0000 deg min^–1^, sampling pitch 0.0200 deg), with a scanning range (2θ) of 2–80° and use of three slits: divergent slit (1 deg), dispersion slit (1 deg) and receiving slit 0.3 (mm).

The X-ray diffraction patterns of the samples were refined using the MAUD software (Materials Analysis Using Diffraction). This program enables a detailed analysis of the crystalline structure through the Rietveld refinement method, allowing for the accurate determination of parameters such as crystallite size, lattice microstrain, lattice parameters, and agreement factors. The use of MAUD ensured an appropriate modeling of the experimental data, contributing to the understanding of the structural characteristics of the silver nanoparticles in the different matrices analyzed.

### 2.5 Analysis of Electrokinetic Potential

The zeta potential (ζ), apparent hydrodynamic size (d), and polydispersity index (PDI) of the AgNPs were analyzed in an aqueous suspension. For sample preparation, the newly synthesized AgNP suspensions were kept in beakers covered with aluminum foil for photoprotection, then transferred to similarly covered Falcon tubes. The samples were centrifuged at 3500 rpm for 15 min. The resulting supernatant was then transferred to new aluminum-foil-covered tubes to eliminate interference from the polymer precipitate and possible process impurities. A 1:10 (v/v) dilution was prepared from the centrifuged suspensions to a final volume of 1 mL for analysis. The apparent hydrodynamic size and PDI were determined by dynamic light scattering (DLS).^5^

### 2.6 Atomic Force Microscopy (AFM)

For analysis, the samples were diluted in a ratio of 1:10 (v/v). Subsequently, a 10 μL aliquot was removed from each sample and deposited on a previously cleaved mica surface. The material was dried for 24 h in a desiccator. The equipment used for the analysis was the TT-AFM in intermittent contact mode, using AFM Silicon tips (ACLA-10 W) at an amplitude frequency of approximately 260 kHz. The Gwyddion 2.67 program was used to process the images.^7^

### 2.7 Thermogravimetry (TGA) and Differential Scanning Calorimetry (DSC)

The curves were obtained using a DTG-60H thermogravimetric analyzer equipped with TA60 software, under a nitrogen atmosphere at a flow rate of 50 mL min^–1^, with a heating rate of 20 °C/min, over a temperature range of 30–800 °C. A sample mass of approximately 3 mg (±0.1 mg) was placed in an alumina crucible. The instrument was calibrated using a standard sample of calcium oxalate monohydrate to verify temperature and mass accuracy.

The curves were obtained using a DSC-60 Plus Differential Scanning Calorimeter, under a nitrogen atmosphere at a flow rate of 50 mL min^–1^, with a heating rate of 10 °C/min, over a temperature range of 30–300 °C. The samples, consisting of approximately 3 mg (±0.1 mg) of the drug, were placed in hermetically sealed aluminum pans. Indium and zinc were used to calibrate the temperature scale and enthalpy response.

### 2.8 Antibacterial Activity

The antibacterial activity of PMMA/CG copolymer, AgNP- PMMA/CG, AgNP-CG and AgNO_3_ was evaluated by determining the minimum inhibitory concentration (MIC) against three bacterial strains: *Staphylococcus aureus* ATCC 29213, *Escherichia coli* ATCC 25922 and *Pseudomonas aeruginosa* ATCC 27853.

#### 2.8.1 Cultivation of Bacterial Strains

The bacteria were grown in Petri dishes containing Mueller–Hinton agar under aerobic conditions, in a bacteriological incubator at 35 ± 2 °C, for 24 h. After this period, and according to the standards recommended,^18^ a bacterial suspension in sterile saline solution (0.85% w v^–1^ NaCl) was prepared to obtain a turbidity standard corresponding to 0.5 on the McFarland scale. (1 to 2.0 × 10^8^ CFU mL^–1^). This suspension was established from an absorbance standard between 0.08 and 0.13, in a UV–visible spectrophotometer, at a wavelength of 625 nm. Once standardized, the suspension obtained was used to prepare the bacterial inoculum used in the execution of the MIC determination protocols^18^

#### 2.8.2 Determination of Minimum Inhibitory Concentration (MIC) and Minimum Bactericidal Concentration (MBC)

The antibacterial potential was evaluated by means of the MIC determination method, using the broth microdilution technique. The bacterial inoculum previously standardized to the 0.5 McFarland scale was diluted in Mueller–Hinton broth, obtaining a final concentration of 5 × 10^5^ CFU mL^–1^.^18^ Using a 96-well microplate, the compounds were subjected to a serial dilution of a ratio of two, with the antibacterial activity being analyzed against the three bacterial strains/plate (representative diagram in the Supporting Information).

For nanoparticles and AgNO_3_, the concentrations ranged from 250 to 1.95 μ mol L^–1^, and the assay was performed according to the,^18^ with some adaptations.^19^ For PMMA/CG, the concentrations ranged from 0.5 to 0.003% of the polymer. The plates were kept aerobically in a bacteriological incubator at 35 ± 2 °C for 24 h, and the lowest concentration capable of inhibiting bacterial growth was defined as the MIC. Following the incubation period and determination of the MIC, the contents of the wells with concentrations equal to or greater than the MIC were subcultured onto Mueller–Hinton agar plates. This was done to determine the minimum bactericidal concentration (MBC) and characterize the effects of the studied compounds as either bactericidal or bacteriostatic.

### 2.9 Cytotoxicity

The RAW 264.7 cell line was grow n in culture flasks using DMEM medium, added with 1% antibiotics and supplemented with 10% fetal bovine serum (FBS) and incubated at 37 °C in an atmosphere with 5% CO_2_. After 24 h of cell incubation, samples were added to the wells at different concentrations (0.5 mmol L^–1^–1.5 × 10^–2^ mmol L^–1^) and incubated for 72 h. The culture medium (DMEM) was used as a negative control, and the cytotoxic activity was measured by the MTT salt reduction method (3-(4,5-dimethylthiazol-2-yl)-2,5-diphenyl tetrazolium bromide). Three hours before completing the incubation time, 25 μL of MTT were added to each well (5 mg mL^–1^) and, subsequently, the culture medium with MTT was aspirated and 100 μL of DMSO were added to each well to dissolve the formazan crystals.^20^ The absorbance was measured by spectrophotometry in a microplate reader at a wavelength of 560 nm.

Cell viability and statistical analyses were performed using GraphPad Prism 8.1. The results are presented as the mean and standard deviation of sextuplicate tests. The experiments were conducted at the BIOFARMATOX laboratory, Department of Antibiotics, UFPE.

## 3 Results and Discussion

### 3.1 FTIR and XRD of Polymers

Figure 1 shows the specific signal of MMA unsaturation between sp^2^ carbons with stretching at 1620 cm^–1^ can be observed. The formation of PMMA from MMA in the FTIR is confirmed by the disappearance of the MMA sp^2^ carbon unsaturation band, since, in PMMA, there is a loss of unsaturation due to the successive addition reactions that form the polymer chain.^21^

**Figure 1:**
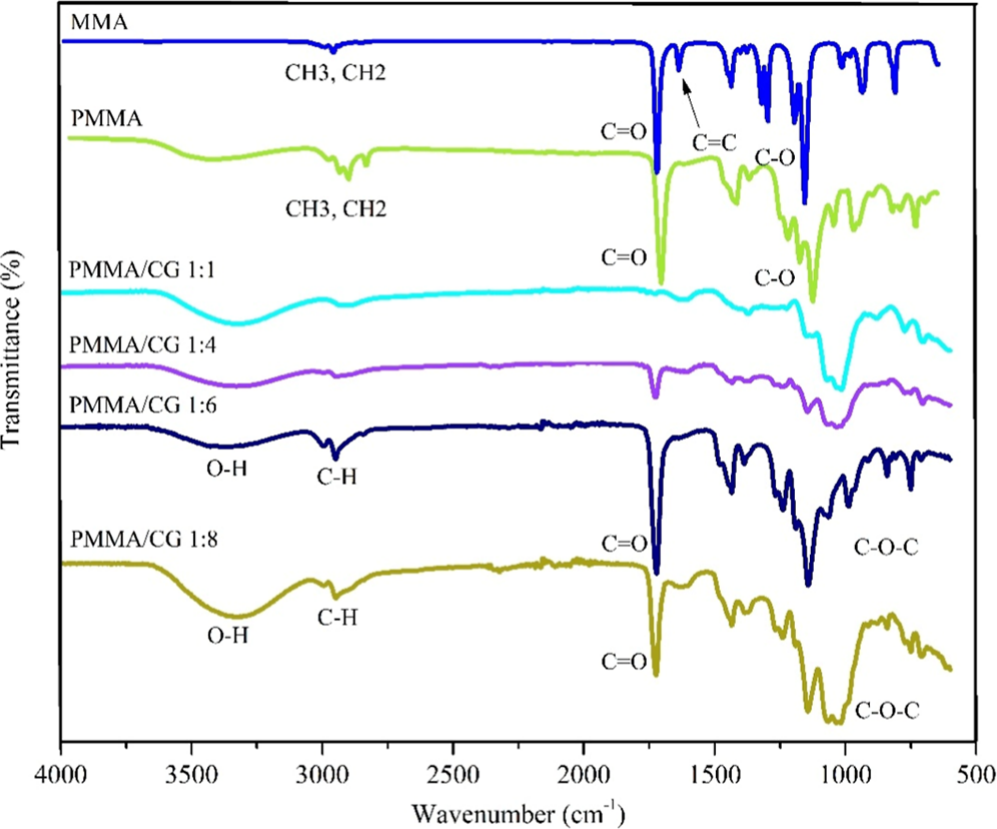
Fourier transform infrared spectrum (FTIR) for MMA, PMMA and PMMA/CG. PMMA/CG: PMMA/*Cashew gum* copolymer; MMA: methyl methacrylate; PMMA: poly­(methyl methacrylate).

Figure 1 also shows the spectra of the PMMA/CG copolymer at ratios of 1:1, 1:4, 1:6, and 1:8, where the hydroxyl and glycosidic bonds are prominent. The O–H stretching is observed at 3200 cm^–1^, while the glycosidic bond is indicated by a stretching signal at 1050 cm^–1^.^22^

In the PMMA/CG copolymer, the C=O stretching of the ester carbonyl, a characteristic of PMMA, is also observed at 1723 cm^–1^. From the spectra, the 1:6 (m/m) ratio showed a greater intensity for the C=O band at 1723 cm^–1^, which indicates better copolymer formation. Consequently, this 1:6 ratio was selected for the AgNP biosynthesis. A schematic representation of the PMMA/CG polymerization can be found in the Supporting Information.

The XRD patterns of the isolated polymers (Figure 2) showed an amorphous structure, with broad signals from 2θ = 10° to 20° for PMMA and 2θ = 20° to 30° for CG. For the PMMA/CG copolymer, the characteristic signals of both polymers were maintained, which corresponds to the overlapping of their polymer chains while preserving the amorphous nature of the materials.

**Figure 2:**
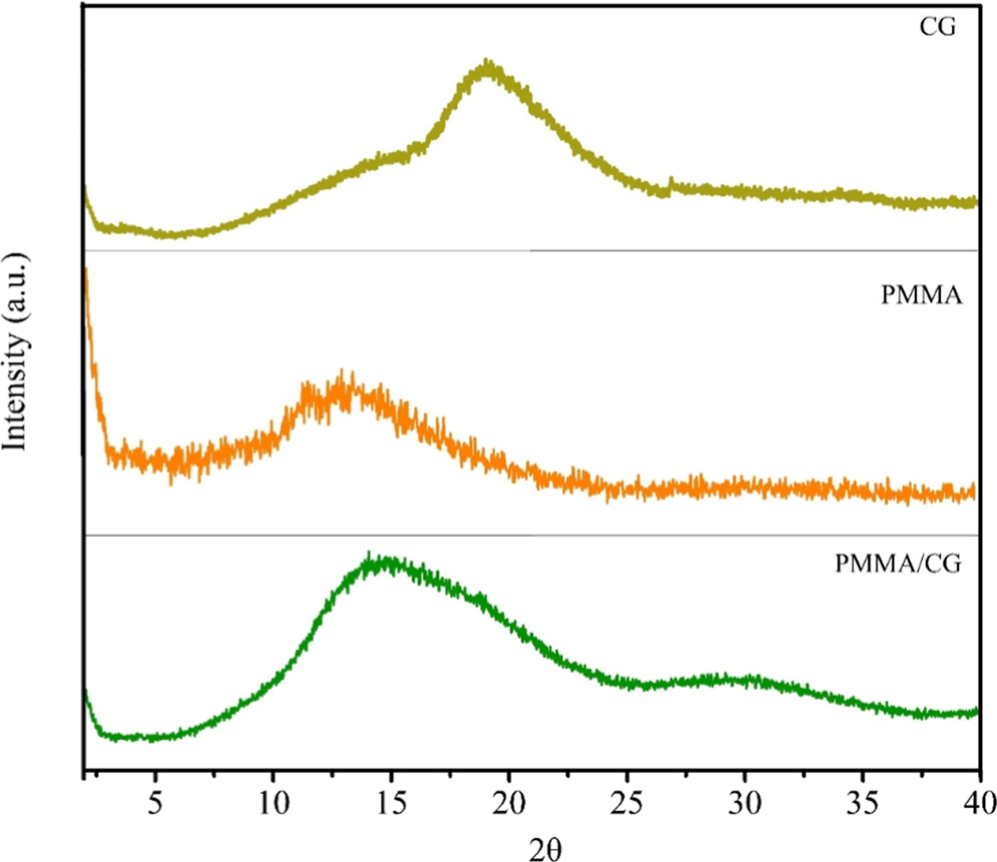
(a) XRD of CG, PMMA and PMMA/CG. CG: *Cashew gum*, PMMA: poly­(methyl methacrylate), PMMA/CG: *Cashew gum*/PMMA copolymer.

### 3.2 FTIR and XRD of Nanoparticles

The FTIR spectra of the biosynthesized AgNPs (Figure 3) show a reduction in the intensity of the –OH band. This suggests that AgNP-PMMA/CG and AgNP-CG are stabilized through the formation of noncovalent inter- and intramolecular interactions between the –OH groups in the polymer solution and the Ag^+^ cations.^4,5,23^ Furthermore, the absence of the C=O signal at 1723 cm^–1^ suggests that the C=O group of the copolymer may also be involved in the formation of AgNPs, similar to the –OH groups.^6^

**Figure 3:**
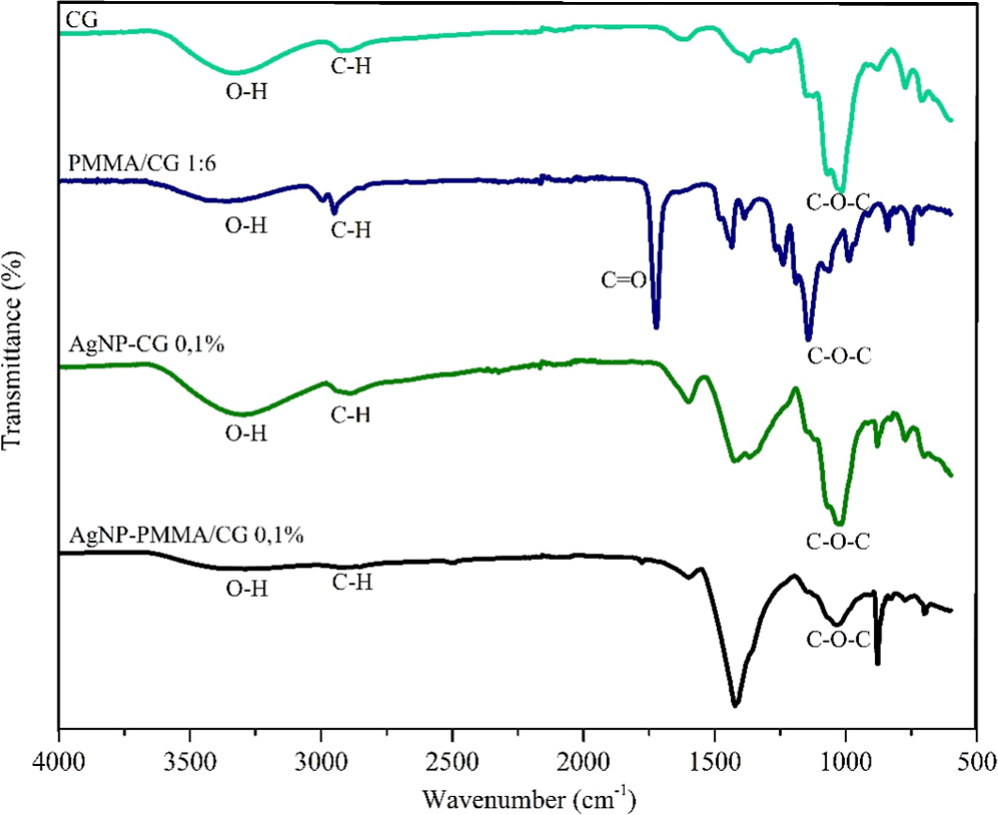
Fourier transform infrared spectrum (FTIR) for CG, PMMA/CG, AgNP/CG and AgNP/CG-PMMA. AgNP/CG: silver nanoparticles obtained from *Cashew gum*; AgNP/CG-PMMA: silver nanoparticles obtained with *Cashew gum*/PMMA copolymer.

The mechanism for AgNP formation is proposed to involve the reduction of Ag^+^ cations. This reduction occurs via the electron pairs of the –OH and C=O functional groups of CG and PMMA/CG, respectively. As these groups are highly nucleophilic, their high electron density facilitates an approach with the metal cation (Ag^+^), leading to its reduction and the nucleation process. This also suggests that both CG and PMMA/CG form a matrix that aids in stabilizing the AgNPs. This mechanism is similar to those reported for other polymers with a chemical structure like CG, such as *Gum arabic* and *Guar gum*.^23,24^ However, the weakening or disappearance of the signal may also be attributed to other factors, such as the physical shielding of the polymeric groups by the AgNPs or a strong interaction between the AgNPs and the polymeric matrix, leading to a change in their vibrational modes.

The absence of the nitrate ion (NO_3_^–^) signal at 1250 cm^–1^ is another indication of AgNP formation. This signal is present in the aqueous AgNO_3_ solution used as the source of Ag^+^ cations. During synthesis, the Ag^+^ cations are reduced and undergo nucleation to form AgNPs; following their formation, the nitrate ion can no longer be detected.^25^

The greater participation of the PMMA C=O group in AgNP-PMMA/CG suggests that its presence in the copolymer works as a more effective reducing agent compared to AgNP-CG. This finding is in agreement with reported literature, where CG is described as a good stabilizing agent but a weak reducing agent.^22^ It also indicates that AgNP-PMMA/CG may exhibit a more stable structure than AgNP-CG.

### 3.3 UV–vis and Electrokinetic Potential of Nanoparticles

Literature data indicate that the size and nucleation of AgNPs are influenced by the concentration of the polymer used as a reducing agent, as well as by the pH and temperature during synthesis.^26–29^ Thus, the type of polymer or copolymer used for AgNP formation may affect the size and stability of the nanoparticles. This is attributed to the concentration of hydroxyl and carboxyl groups, which favor the reduction of Ag^+^ to Ag^0^.^5^

The UV–vis spectra of the AgNPs prepared from the PMMA/CG copolymer (at a 1:6 w/w ratio) and from pure CG (Figure 4) show the characteristic surface plasmon resonance (SPR) band between 400 and 430 nm, confirming AgNP formation.^30^ Furthermore, the absence of significant absorption in the region between 450 and 800 nm suggests that the AgNP-PMMA/CG composite is well-dispersed with little aggregation.^31^ This indicates that both the PMMA/CG copolymer and CG are suitable stabilizing agents for the biosynthesis of AgNPs.^7^

**Figure 4:**
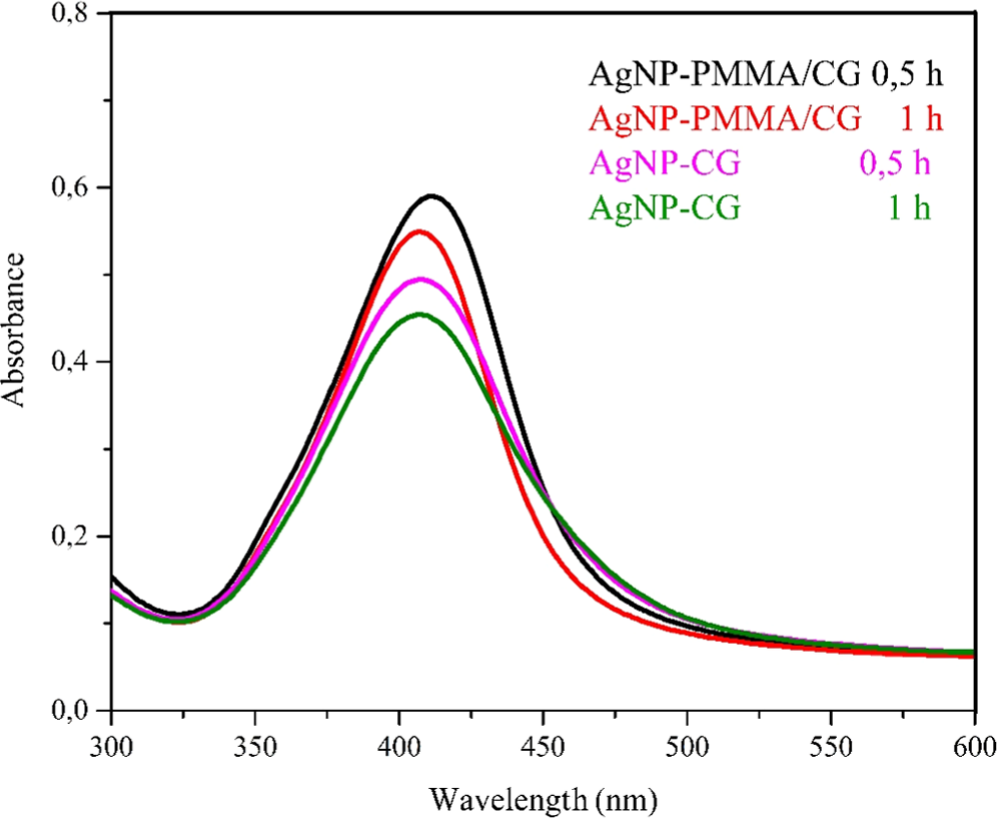
UV–vis spectrum of AgNP-CG and AgNP -PMMA/CG. AgNP-CG: silver nanoparticles obtained from *Cashew gum*; AgNP/CG-PMMA: silver nanoparticles obtained with *Cashew gum*/PMMA copolymer.

In conjunction with the UV–vis data (Figure 4), the DLS results allowed for the evaluation of the formation, size, and relative colloidal stability of the AgNPs (Table 1). The data for AgNP-PMMA/CG were particularly promising, presenting a smaller size, a negative surface charge, high colloidal stability, and a low PDI that indicated good dispersion, a significant finding for particles generated by biosynthesis.

**Table 1:** Size, Zeta Potential and PDI of AgNP -CG and AgNP -PMMA/CG^a^

AgNP	size (nm)	PDI	zeta (mV)
AgNP -PMMA/CG	40.81 ± 1.04	0.46 ± 0.008	–36.36 ± 1.25
AgNP -CG	71.16 ± 2.5	0.71 ± 0.024	–28.93 ± 9.8

a AgNP -CG: silver nanoparticles obtained from *Cashew gum*; AgNP/CG-PMMA: silver nanoparticles obtained with *Cashew gum*/PMMA copolymer.

These results are consistent with literature data,^5^ in which the synthesis of AgNPs from modified CG (carboxymethylated *cashew gum*) was found to promote a reduction in hydrodynamic size and a more negative zeta potential compared to AgNPs obtained from unmodified CG. Furthermore, a comparison of our data for AgNP-PMMA/CG and AgNP-CG with those from the literature^5^ shows that our synthesized nanoparticles have more negative zeta potentials and smaller hydrodynamic sizes. These findings indicate that the PMMA/CG copolymer is a promising agent for the reduction and stabilization of AgNPs, as it promotes greater colloidal stability and size reduction. These characteristics are important for interactions with biomolecules in biological applications.

Although medium and long-term monitoring are necessary to confirm this result, it can be inferred that the stabilization mechanisms of AgNP-PMMA/CG include electrostatic repulsion and steric hindrance. The electrostatic repulsion is generated by the charges on the reduced silver, while the steric hindrance is caused by the intertwined polymeric chains of CG and PMMA, which work together to prevent particle aggregation.^5,6^

### 3.4 XRD and Rietveld Refinement of Nanoparticles

For both AgNPs, diffraction peaks were observed corresponding to the Miller indices (*hkl*) for AgNP (111), (200), (220), and (311), with indexed values in agreement with the standard reference (Ag—ICSD 22434), as shown in Figure 5. The high degree of crystallinity of these peaks demonstrates the quality and well-defined structure of the AgNPs, confirming the successful biosynthesis using PMMA/CG and CG. In addition to the Bragg peaks, the XRD patterns of the AgNPs showed additional unassigned peaks, which may be attributed to the biopolymeric material used in the biosynthesis, as also reported in the literature.^32^

**Figure 5:**
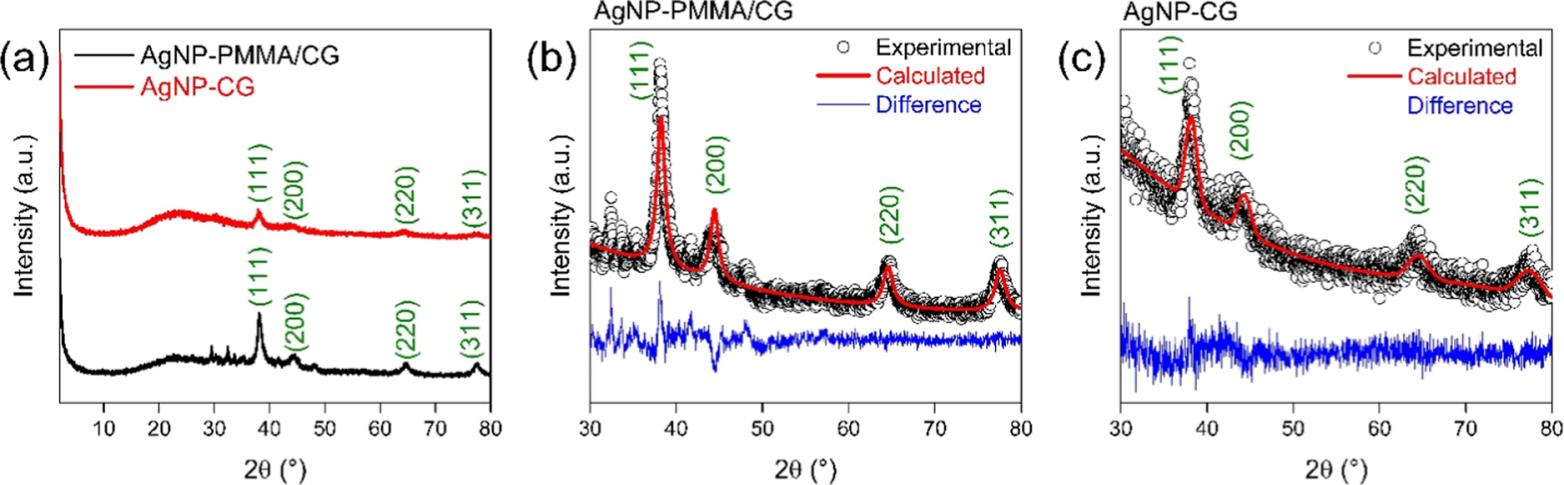
(a) XRD of AgNP -CG and AgNP -PMMA/CG. (b,c) XRD patterns (AgNP -CG-PMMA and AgNP -CG) with Rietveld refinement. AgNP/CG: silver nanoparticles obtained from *Cashew gum*; AgNP/CG-PMMA: silver nanoparticles obtained with *Cashew gum*/PMMA copolymer.

Both AgNPs exhibited the most intense peak for the (111) plane, this plan is characteristic of materials with a face-centered cubic (FCC) structure. Furthermore, the intensity ratios of the diffraction peaks, (200)/(111) and (220)/(111), for AgNP-PMMA/CG and AgNP-CG (as shown in Table 2) were close to the theoretical values, indicating a good correlation between the theoretical and experimental data.^33^

**Table 2:** Intensity Ratio between Diffraction Peaks for AgNP-CG and AgNP -PMMA/CG^a^

experimental diffraction ratio	AgNP -GC	AgNP-PMMA/GC	convencional value^b^
*I*_(200)/(111)_	0.24	0.21	0.31
*I*_(220)/(111)_	0.25	0.20	0.22

a AgNP/CG: silver nanoparticles obtained from *Cashew gum*; AgNP/CG-PMMA: silver nanoparticles obtained with *Cashew gum*/PMMA copolymer.

b^32^.

The original XRD analysis data has undergone a refinement process, in which the experimental diffraction data is compared with the ICSD standard, the output information contains details about the crystal structure parameters. Table 3 presents the results of the structural refinement of silver nanoparticles (AgNP) incorporated into different matrices: AgNP -CG-PMMA and AgNP -CG, in addition to the reference standard Ag—ICSD 22434. The Table 3 contains the main crystallographic parameters and concordance factors obtained through the Rietveld refinement method.

**Table 3:** Crystallographic Planes and Parameters of Silver Nanoparticles AgNP -PMMA/GC and AgNP -CG^a^

	crystallographic parameters			agreement factors		
sample	size (nm)	*a* (Å)	ε	*R*_wp_ (%)	*R*_exp_ (%)	χ^2^
Ag—ICSD 22434		4.032(4)				
AgNP -CG-PMMA	11	4.0883(1)	0.01	11.16	7.13	1.56
AgNP -CG	7.24	4.0865(6)	0.015	8.49	7.61	1.11

a Size (nm): crystallite size; *a*: lattice parameter in angstromAngstrom; ε: microstrain; *R*_wp_ (%): Weighted Profile *R*-factor; *R*_exp_ (%): expected *R*-factor; χ2: (Chi-squared).

Regarding the size of the crystallites, it is observed that the AgNP -GC-PMMA sample presents larger particles (11 nm) compared to AgNP -GC (7.24 nm). This increase in size can be attributed to the presence of PMMA, which possibly acts as a stabilizing or growth-directing agent, favoring the formation of larger particles.

The lattice parameter “*a*” is slightly expanded in the synthesized samples in relation to the reference value (4.032(4) Å), with values of 4.0883(1) Å for AgNP -CG-PMMA and 4.0865(6) Å for AgNP -CG. This expansion may be associated with the presence of internal stresses or structural defects, common in nanocrystalline materials. In fact, the associated microstrain of AgNP-CG is larger (0.015) than AgNP-CG-PMMA (0.01) (see Table 3).

As for the agreement factors, it is observed that the AgNP -CG sample presented the best results, with a lower *R*_wp_ value (8.49%) and a lower χ^2^ value (1.11), indicating a more accurate fit of the theoretical model to the experimental data. In contrast, the sample with PMMA presented *R*_wp_ of 11.16% and χ^2^ of 1.56, reflecting a lower quality of fit. The *R*exp values were similar for both samples, confirming that the applied model was appropriate.

In summary, the AgNP -CG sample demonstrated better crystalline quality and more reliable structural adjustment, in addition to presenting smaller particles. On the other hand, the use of PMMA seems to favor the growth of larger particles, although with a slight loss in the accuracy of the structural adjustment.

### 3.5 Thermal Analysis and AFM of Nanoparticles

The first event observed for the PMMA/CG copolymer (Figure 6) occurs up to 100 °C and corresponds to the loss of hydration water (10%). The second event, with a DTA peak at 260 °C, is attributed to the condensation of hydroxyl groups present in both CG and PMMA. The third event, with a DTA peak at 300 °C, corresponds to the thermal degradation of the CG polysaccharide structure,^6^ as well as to steps related to the depolymerization of PMMA involving the scission of unsaturated terminal groups. The final events, occurring between 320 and 450 °C,^17^ correspond to the breakdown of PMMA chains, leading to the complete loss of the organic structure with the release of CO_2_ and H_2_O.

**Figure 6:**
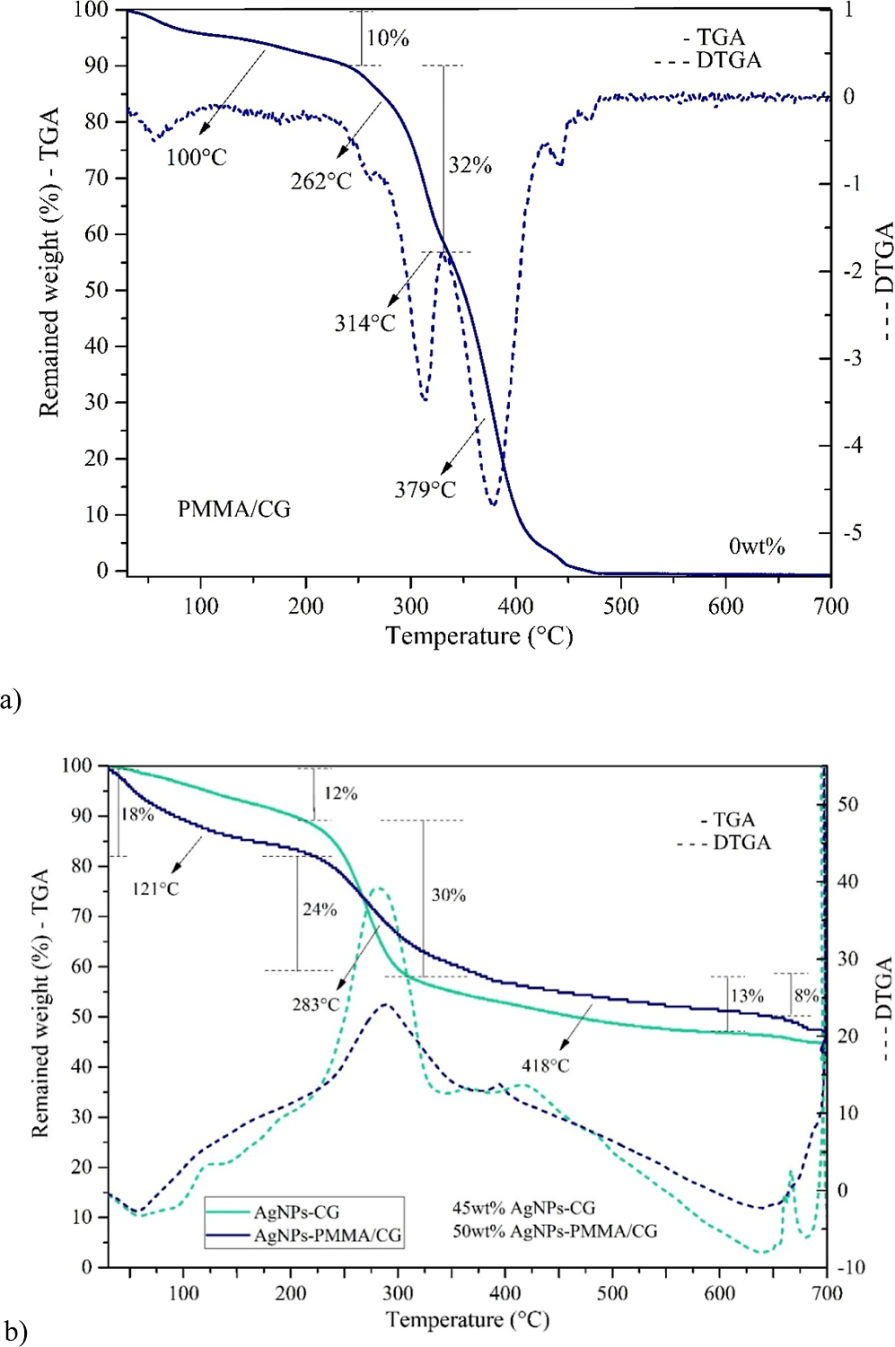
Thermogravimetry of (a) PMMA/CG and (b) AgNP-PMMA/CG and AgNP-CG. AgNP-PMMA/CG: silver nanoparticles obtained with *Cashew gum*/PMMA copolymer, AgNP-CG: silver nanoparticles obtained from *Cashew gum*.

For the nanoparticles (Figure 6), events associated with the presence of CG and PMMA are maintained in AgNP-CG/PMMA, while events related to PMMA depolymerization are absent in AgNP-CG. Due to the higher number of hydroxyl groups present in AgNP-CG/PMMA, greater mass loss is observed in the initial events (up to 300 °C), as the glycosidic structure decomposes more rapidly compared to the unsaturated groups in AgNP-CG/PMMA, indicating increased thermal stability in this temperature range. At the end of the process, a considerable amount of residue remains compared to the CG/PMMA copolymer: 45% for AgNP-CG and 50% for AgNP-CG/PMMA, attributed to the presence of silver in the nanoparticles.

In the DSC analysis (Figure 7), the glass transition of the copolymer is observed.^17^ For AgNP-PMMA/CG, a more defined peak is observed, indicating greater organization or crystallinity compared to AgNP-CG. This is consistent with the XRD and AFM data and suggests that the addition of AgNPs has a greater impact on the structural organization of the polymers than on their overall thermal properties. However, more in-depth analyses, such as the determination of activation energy (*E*_a_) or the quantification of mass loss from specific fragments in the TGA, could provide further insights into the thermal stability of the AgNPs.

**Figure 7:**
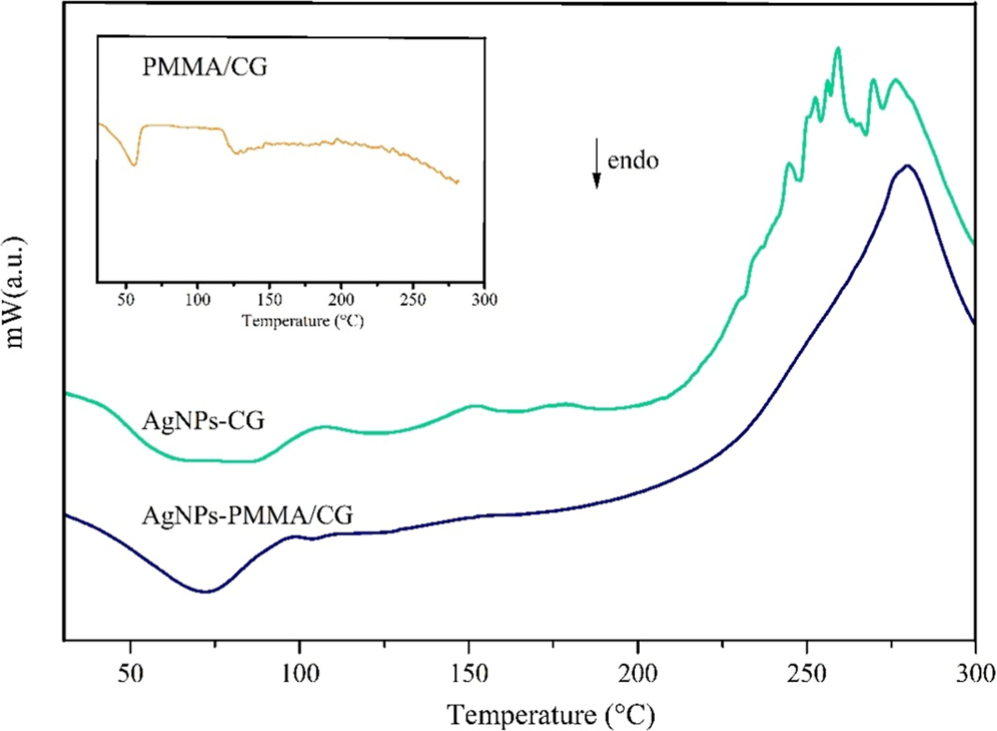
Differential Scanning Calorimetry of AgNP-PMMA/CG e AgNP-CG. AgNP-PMMA/CG: silver nanoparticles obtained with *Cashew gum*/PMMA copolymer, AgNP-CG: silver nanoparticles obtained from *Cashew gum*.

The AFM data (Figures 8 and 9) confirmed the better structural organization of the AgNP-PMMA/CG, as evidenced by its more spherical shape and defined edges. These findings were corroborated by the FTIR and XRD analyses. Furthermore, the size distribution (Figure 10) indicated a smaller size and less heterogeneity for AgNP-PMMA/CG compared to AgNP-CG, which agrees with the DLS data. Thus, these characterization results collectively indicated that AgNP-PMMA/CG was the most suitable system for evaluating antimicrobial activity.

**Figure 8:**
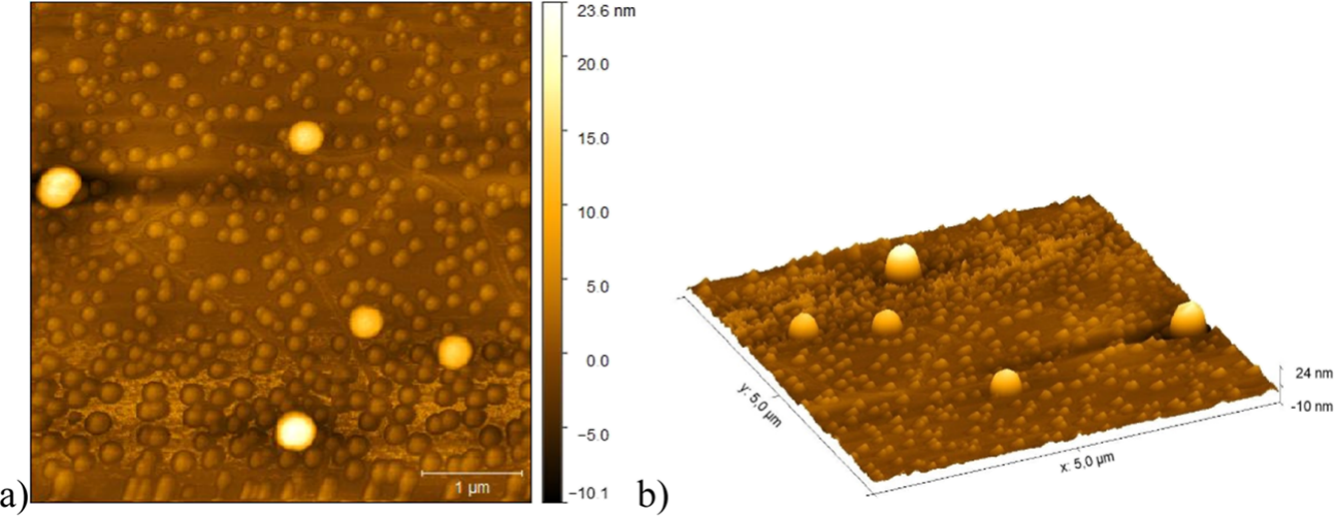
AFM of AgNP-PMMA/CG. (a) 2D image of a 5 × 5 μm area and (b) 3D image of a 5 × 5 μm area. Both images were acquired at a resolution of 512 × 512 pixels. AgNP/PMMA-CG: silver nanoparticles obtained with *Cashew gum*/PMMA copolymer.

**Figure 9:**
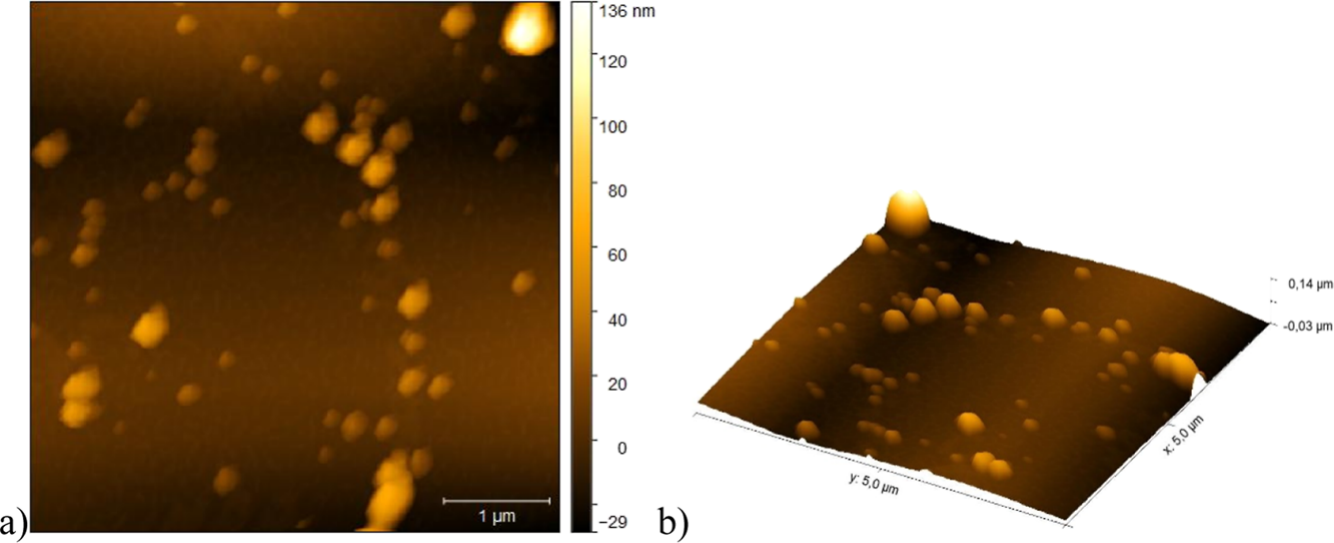
AFM of AgNP-CG. (a) 2D image of a 5 × 5 μm area and (b) 3D image of a 5 × 5 μm area. Both images were acquired at a resolution of 512 × 512 pixels. AgNP-CG: silver nanoparticles obtained from *Cashew gum*.

**Figure 10:**
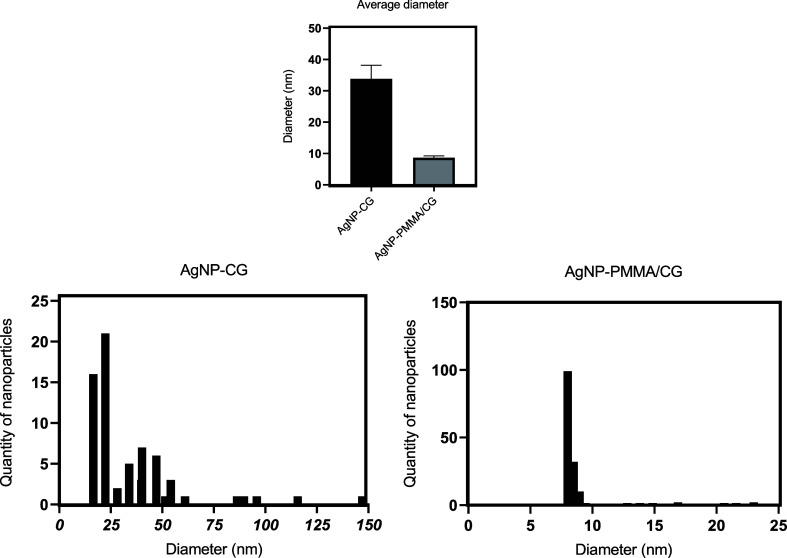
Size distribution of AgNP-CG and AgNP-PMMA/CG based on AFM images. AgNP-CG: silver nanoparticles obtained from *Cashew gum*.

### 3.6 In Vitro Studies: Antimicrobial Activity and Cytotoxicity for Nanoparticles

The MIC (minimum inhibitory concentration) of a bacterium is the lowest concentration capable of inhibiting the growth of a bacterial inoculum in a standard test. In contrast, the MBC (minimum bactericidal concentration) is the lowest concentration of an antibiotic that kills the inoculum and is determined from broth dilution MIC tests by subculturing onto antibiotic-free agar medium.^34^

*S. aureus* and *E. coli* are two bacterial species with etiological importance in human toxic and infectious diseases. *S. aureus* is a Gram-positive coccus that causes endogenous infections, such as those in immunocompromised patients, and produces heat-stable toxins that can remain in food even after cooking. *E. coli*, a Gram-negative bacillus commonly found in the intestinal microbiota of animals, is associated with various human infections, including urinary tract infections, sepsis, neonatal meningitis, and gastroenteritis.^35–37^

*P. aeruginosa* is another pathogenic, opportunistic, Gram-negative bacterium. One of its main virulence factors is its ability to form an adhesive extracellular matrix, which leads to biofilm formation. This biofilm is a viscous surface where microorganisms adhere to protect themselves from unfavorable environmental conditions.^38,39^

For the antimicrobial assays, the PMMA/CG polymer showed no bacteriostatic or bactericidal activity for the strains tested (Table 4). These results agree with those reported in the literature, where the polymer alone also showed no antibacterial activity at the concentrations tested (*Cashew gum* and *acetylated cashew gum* > 750 μg/mL, for *S. aureus* ATCC and *E. coli* ATCC).^5,40^

**Table 4:** Minimum Inhibitory Concentration (MIC) and Minimum Bactericidal Concentration (MBC) of the Compounds Studied^a^ ^,^ ^b^

	PMMA/CG		AgNP -PMMA/CG		AgNP-CG		AgNO_3_	
bacterial strains	MIC	MBC	MIC	MBC	MIC	MBC	MIC	MBC
S. aureus ATCC 29213	>0.5	NT	250 μmmol L^–1^	>250 μmmol L^–1^	>250 μmmol L^–1^	NP	15.62 μmmol L^–1^	31.25 μmmol L^–1^
E. coli ATCC 25922	>0.5	NT	250 μmmol L^–1^	>250 μmmol L^–1^	250 μmmol L^–1^	>250 μM	62.5 μmmol L^–1^	62.5 μmmol L^–1^
P. aeruginosa ATCC 27853	>0.5	NT	62.5 μmmol L^–1^	>250 μmmol L^–1^	250 μmmol L^–1^	>250 μM	31.25 μmmol L^–1^	62.5 μmmol L^–1^

a The results are expressed in μmmol L^–1^ of silver for AgNP- PMMA/CG, AgNP-CG and AgNO_3_ and in percentage for the PMMA/CG polymer.

b NT = not tested. The results are expressed in μM of silver for AgNP and AgNO_3_ and in percentage for the PMMA/CG polymer. AgNP-PMMA/CG: silver nanoparticles obtained with *Cashew gum*/PMMA copolymer. AgNP-CG: silver nanoparticles obtained with *Cashew gum*. NP: not performed.

However, the AgNP-PMMA/CG and AgNP-CG showed significant results at the concentrations tested. The AgNP-PMMA/CG exhibited an MIC ranging from 62.5–250 μmmol L^–1^ of silver and an MBC higher than 250 μmmol L^–1^ of silver, while AgNP-CG showed MICs equal to 250 μmmol L^–1^ and MBC higher than 250 μmmol L^–1^ for *E. coli* and *P. aeruginosa* (Table 4). These results indicate an antibacterial effect, characterized as bacteriostatic for both nanoparticles. Furthermore, a lower MIC was observed for AgNP-PMMA/CG against *P. aeruginosa*, suggesting possible action against biofilm-forming bacteria, a key mechanism of bacterial resistance.

Despite these positive results, it is observed that the AgNPs produced by the traditional AgNO_3_ reduction method, show greater activity against all bacteria tested compared to AgNP-CG and AgNPs-PMMA/CG, demonstrating better performance (Table 4).

AgNPs produced by the traditional method have a higher percentage of silver in their composition in relation to those obtained by biosynthesis, and the Ag ions are responsible for their inherent antimicrobial activity.^3^ Literature data^5,6,40,41^ also indicates lower MIC values for AgNPs obtained by biosynthesis when compared to their respective polymers alone: AgNO3 (13.5 μgAg/mL and 3.37 μgAg/mL in tests with *S. aureus* and *E. coli*, respectively), compared to the results obtained by biosynthesized AgNP (14.05 μgAg/mL and 3.51 μgAg/mL in tests with *S. aureus* and *E. coli*, respectively), which confirms that the presence of silver is essential for the antimicrobial activity.^6^

The greater susceptibility of Gram-negative bacteria to AgNPs may be due to the rapid internalization of the nanoparticles through their thin, peptidoglycan-poor cell walls. This process inactivates and/or alters protein structure, leading to cell death. In contrast, Gram-positive bacteria have cell walls with a thick peptidoglycan layer, which makes it more difficult for AgNPs to be internalized into the cytoplasm. This may explain the need for a higher concentration of the agent for antibacterial action.^41^

However, the traditional chemical synthesis of AgNPs has several disadvantages, such as high energy consumption, expensive equipment, and the use of compounds that are toxic to humans and the environment.^4^ Therefore, the search for the development of silver nanoparticles through biosynthesis, such as AgNP-CG and AgNPs-PMMA/CG, lies in balancing the maintenance of their antimicrobial action with the reduction of toxicity, in order to enable a formulation with safe therapeutic use.

In general, the antibacterial activity of AgNPs occurs due to the interference of silver with the bacterial bioenergetic pathway, possible mechanisms of action are as follows: Due to their small size, AgNPs can be internalized by the cell membrane, where they release Ag^+^ cations. These cations interact with the sulfhydryl groups of amino acids, inhibiting enzymes involved in the electron transport chain, this blocks energy synthesis and inactivates the bacterial cell by causing protein precipitation. In addition, the Ag^+^ cations released from the internalized AgNPs can also induce oxidative stress by forming reactive oxygen species. This causes dysfunction and disorganization in the structure of DNA, lipids, and proteins, eventually leading to cell death.^42–44^

The interaction between proteins and prata nanoparticles is one of two main toxicity phenomena of prata nanoparticles. Among these interactions, serum proteins, albumin, keratins and Hb (hemoglobin) can be mentioned. For Hb, the complex of Hb–AgNPs is formed, through the interaction between homemade and tryptophan. After ligation, the structure of the β sheet of hemoglobin increases and begins to split, forming a structure enveloped in the electron transfer mechanism.^41–44^

In fibroblasts, AgNPs can break the cell membrane, due to oxidative damage. The influx of calcium generated through damage, allows for potential variation in mitochondrial membranes and overproduction of reactive oxygen species.^41–44^

However, to fully understand the mechanism(s) involved in the cell death promoted by AgNP-PMMA/CG and AgNP-CG, detailed investigations on microbial killing and toxicity to mammalian cells must be performed. In addition to evaluating antibacterial activity, in vitro cytotoxicity assays are often used to characterize the biological response to AgNP, and its results can help identify hazards associated with exposure.

Figure 11 demonstrates the viability of RAW cells exposed to AgNPs for 72 h, it shows that both AgNP-CG and AgNP-PMMA/CG, at the concentrations tested (0.5 mmol L^–1^–1.5 × 10^–2^ mmol L^–1^), presented no significant difference in cell viability compared to the negative control. These results indicate that higher doses are needed to result in significant cellular toxicity, similar results were reported in normal Vero cell lines,^46^ which were exposed to a crude extract of silver nanoparticles where no toxicity was observed at low concentrations, requiring a concentration of 500 μg/mL (CC_50_ of 500 μg/mL).^46^

**Figure 11:**
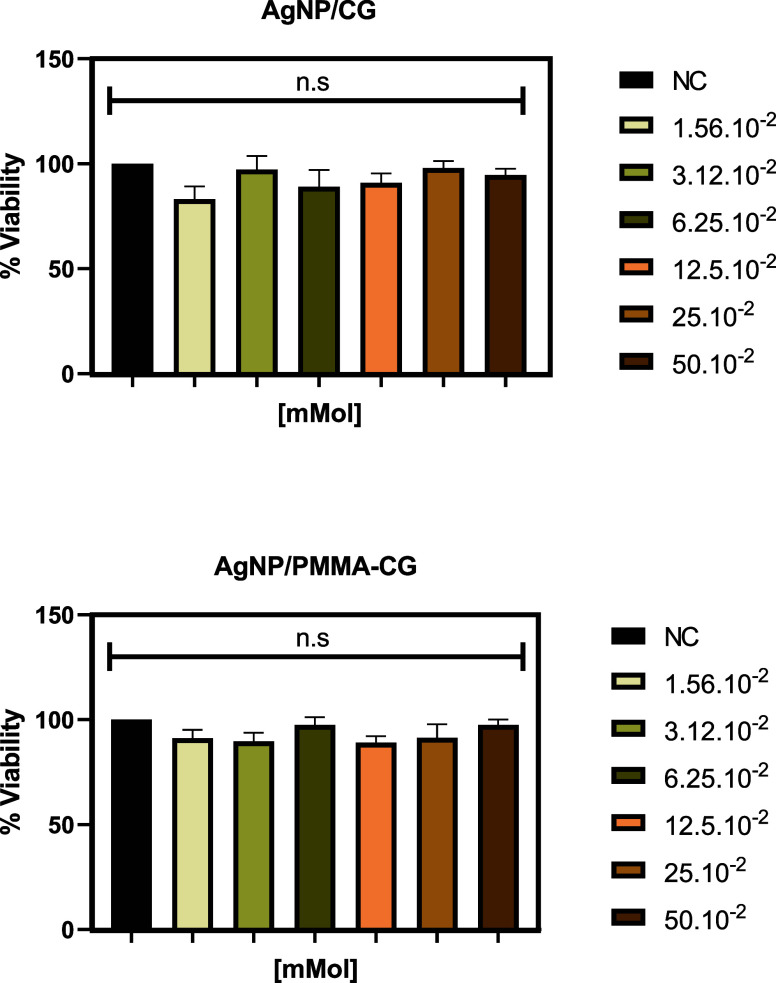
Percentage of cell viability by the MTT method of macrophages (RAW) treated for 72 h with AgNP-CG and AgNP-PMMA/CG. Results expressed by analysis of variance (ANOVA) followed by Tukey’s post-test; ****p < 0,0001. AgNP -CG: silver nanoparticles obtained from *Cashew gum*; AgNP-PMMA/CG: silver nanoparticles obtained with *Cashew gum*/PMMA copolymer; NC: negative control.

This confirms cell viability at all concentrations tested, which, in accordance with ISO 10.993–5,^45^ suggests that biosynthesis produced biocompatible AgNPs with potential therapeutic applications.

## 4 Conclusion

The results showed that in situ polymerization promoted the effective modification of the PMMA/CG biopolymer. The biosynthesis of two types of AgNPs—AgNP-PMMA/CG and AgNP-CG—was revealed by the detection of the surface plasmon resonance band. FTIR analysis indicated that groups participated in the reduction of Ag^+^, suggesting that PMMA/CG is a good reducing agent. AgNP-PMMA/CG acted as a stabilizing or directing agent, favoring crystalline growth. Furthermore, it exhibited greater colloidal stability and better structural organization as shown by AFM. In the antimicrobial activity assay, AgNP-PMMA/CG demonstrated better bacteriostatic action against *P. aeruginosa*, *E. coli*, and *S. aureus* strains, in addition to showing adequate cell viability in murine macrophages. Thus, it can be concluded that the biosynthesis of AgNP-PMMA/CG yielded promising NP due to their biocompatibility, bacteriostatic activity, good structural organization, and more sustainable synthesis method compared to traditional approaches.

## Supplementary Material



## Notes

Contains figures with schematic representation for the polymerization of PMMA/CG and representative scheme for evaluating antimicrobial activity through the microdilution test (PDF)
