# Supplementary material for: Biosynthesis of Silver Nanoparticles from Hybrid Polymer: Characterization, Approach from XRD and Investigation of Antimicrobial Activity
Source: ACS Omega. 2026 Apr 11;11(15):22918–28. doi: 10.1021/acsomega.5c12247 (PMC13103824; doi:10.1021/acsomega.5c12247)

BIOSYNTHESIS OF SILVER NANOPARTICLES FROM HYBRID POLYMER: CHARACTERIZATION, APPROACH FROM XRD AND INVESTIGATION OF ANTIMICROBIAL ACTIVITY

Expedito Lopes Fernandes Júnior[1], Izabel Maria de Melo Amaral[1], George Torres de Lima[2], Raí Emanuel da Silva[3], Alyne Rodrigues de Araújo-Nobre[3], Rafael Alexandre Raimundo[4,5], Luise Lopes Chaves[1], Antônia Carla de Jesus Oliveira[1], Teresinha Gonçalves Silva[2], Mônica Felts de La Roca Soares [1], José Lamartine Soares Sobrinho[1*], Amanda Damasceno Leão[1*]

[1] Quality Control Core of Medicines and Correlates - NCQMC, Department of Sciences, Federal University of Pernambuco - UFPE, Recife, State of Pernambuco, Brazil. CEP: 50670-901

[2] Laboratory of Pharmatoxicological Prospecting of Biological Products, Department of Antibiotics, Federal University of Pernambuco - UFPE, Recife, State of Pernambuco, Brazil. CEP: 50670-901

[3] Biodiversity and Biotechnology Research Center -BIOTEC, Campus de Parnaíba, Parnaíba Delta Federal University- UFDPar, Parnaíba, State of Piauí, Brazil. CEP: 64202-020

[4] Department of Materials Science and Engineering, Federal University of Rio Grande do Norte - UFRN, Natal, State of Rio Grande do Norte, Brazil. CEP: 59078-970

[5] TEMA - Centre for Mechanical Technology and Automation, Department of Mechanical engineering, University of Aveiro, Aveiro, Portugal. CEP: 3810-193

* To whom correspondence should be addressed,
damasceno.leao@gmail.com
jose.ssobrinho@ufpe.br

Figure S1- Schematic representation for the polymerization of PMMA/CG.

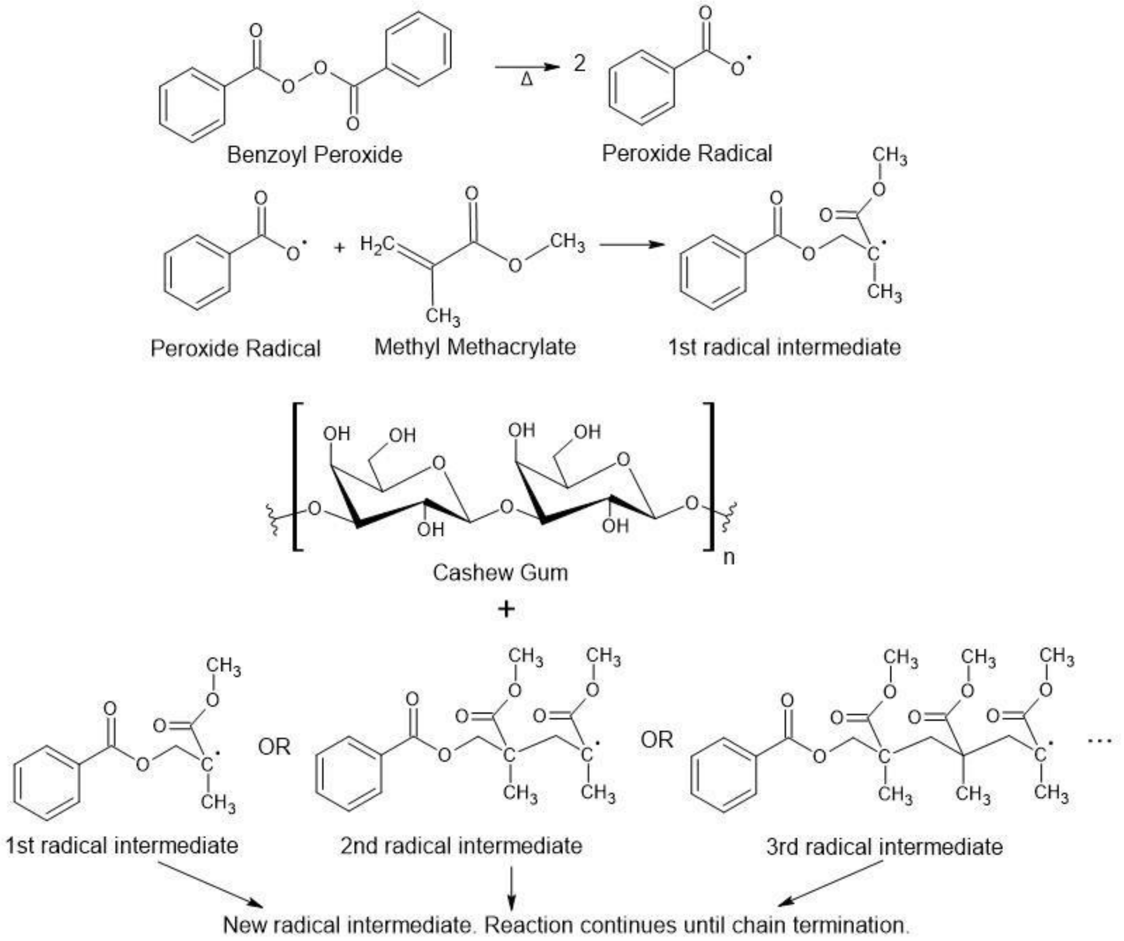

The upper portion of the figure depicts the initiation phase of the radical reaction. The lower portion demonstrates the propagation phase. GC, as a macromolecule, can interact with radicals at any stage of propagation, generating grafts of higher and lower molar mass from the hydrophilic functional groups.

Figure S2- Representative scheme for evaluating antimicrobial activity through the microdilution test.

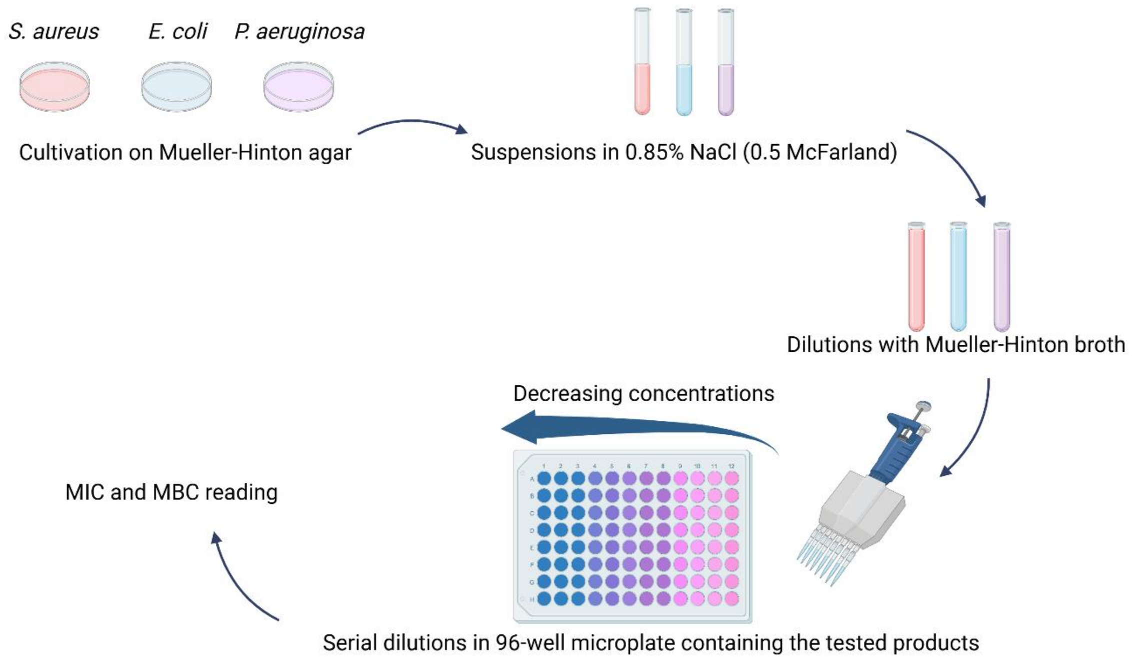

Supplement: Supplementary file 1 [file ao5c12247_si_001.pdf]
